# Kaposi sarcoma initially manifested itself as blindness

**DOI:** 10.1016/j.clinsp.2024.100489

**Published:** 2024-10-16

**Authors:** Ana Claudia Fiorini, Fulvio A. Scorza, Josef Finsterer, Carla A. Scorza

**Affiliations:** a Programa de Estudos Pós-Graduado em Fonoaudiologia, Pontifícia Universidade Católica de São Paulo (PUC-SP), São Paulo, SP, Brazil; b Departamento de Fonoaudiologia, Escola Paulista de Medicina/Universidade Federal de São Paulo (EPM/UNIFESP), São Paulo, SP, Brazil; c Disciplina de Neurociência, Universidade Federal de São Paulo/Escola Paulista de Medicina (UNIFESP/EPM), São Paulo, SP, Brazil; d Neurology Neurophysiology Center, Vienna, Austria

## Correspondence

Kaposi's Sarcoma (KS) is a neoplasm of lymphatic endothelial cells that is probably caused by the Human Herpesvirus-8 (HHV-8).^1^ It can affect the skin and mucous membranes as well as internal organs. KS is a low-grade, infiltrative locally aggressive, rarely metastatic tumor.^1^ In rare cases, KS can be associated with Giant Cell Arteritis (GCA) as a paraneoplastic phenomenon.^2^, ^3^, ^4^, ^5^, ^6^, ^7^, ^8^, ^9^ A complication of GCA is visual impairment, but to our knowledge, no patient with progressive visual impairment leading to blindness as a paraneoplastic complication of KS has been reported.

The patient is an 88-year-old man who had developed bilateral visual impairment four months prior to admission. The ophthalmologic examination and biopsy of the temporal artery revealed an atypical GCA. Despite the administration of glucocorticoids and an initial partial improvement, the visual impairment progressed, and he became functionally blind.

He had a history of dizziness and low blood pressure since a few days before admission, pulmonary embolism two months before admission, non-specific, livid, discolored skin lesions on the back of the right foot since seven months, hypoacusis, hypothyroidism, arterial hypertension, hyperlipidemia, prostatic hyperplasia, omarthrosis, transient ischemic attack and Thrombendarterectomy (TEA) of the right carotid artery at the age of 81 years, and osteoporosis.

The neurological examination revealed a marked impairment of visual acuity, so that only light-dark discrimination was possible, hypoacusis, dysdiadochokinesia and ataxia bilaterally, absent Achilles tendon reflexes, diminished right patellar tendon reflex, mild lower limb ataxia bilaterally, cutaneous lesion over the dorsum of the right foot as well as stance and gait ataxia with a tendency to fall. He denied claudication masticatorica and the temporal arteries were not swollen.

The ECG showed paroxysmal atrial fibrillation. Nerve conduction studies indicated a demyelinating polyneuropathy. Magnetic resonance imaging of the brain showed bilateral extensive White Matter Lesions (WML) and a small right frontal meningioma without evidence of cytotoxic edema or hemorrhage. Magnetic Resonance Angiography (MRA) showed only mild ectasia of the basilar artery with no evidence of vasculitis. Ophthalmologic examination revealed amaurosis on the right side and markedly decreased visual acuity on the left side. On dermatologic examination, cutaneous vasculitis was suspected, but a skin biopsy clearly revealed a KS on the dorsum of the right foot. He was discharged after 10 days with atorvastatin, L-thyroxine, pantoprazole, prednisolone (25 mg/d), amlodipine, bisoprolol, calcium, tamsulosin and rivaroxaban. He received zoledronic acid for KS.

The patient is interesting for GCA, which led to blindness as a secondary, paraneoplastic manifestation of KS. GCS was interpreted as secondary to KS for several reasons. First, the visual impairment developed three months after the appearance of the skin lesions. The presence of skin lesions prior to the onset of visual impairment strongly suggests that the malignancy was the initiating factor rather than the other way around and that there was a causal relationship between the two. Second, the association between KS and GCA has been reported previously.^2^, ^3^, ^4^, ^5^, ^6^, ^7^, ^8^, ^9^ Third, vasculitis is commonly known as a paraneoplastic complication of various malignancies, including colon carcinoma (urticarial vasculitis),^10^ renal cell carcinoma (leukoclastic),^11^ neuroendocrine carcinoma (large vessel),^12^ lung carcinoma (ANCA-associated), squamous cell carcinoma, thyroid carcinoma, and esophageal carcinoma.

The visual impairment was attributed to the GCA after alternative causes such as stroke, hemorrhage, cerebral mass lesion, optic neuritis, Posterior and Anterior Ischemic Optic Neuropathy (PION, AION), uveitis, and retinopathy had been thoroughly ruled out. The vertigo on admission was attributed to arterial hypotension and polyneuropathy on the one hand and the extensive WMLs on the other. The polyneuropathy was also interpreted as paraneoplastic, as alternative causes of polyneuropathy such as diabetes, alcoholism, chemotherapy, genetic causes, vitamin deficiency, poisoning, and autoimmune diseases had been thoroughly ruled out. Although vasculitis can cause polyneuropathy, it was excluded in the present case as GCA has rarely been reported in association with polyneuropathy. The initial arterial hypotension was attributed to the involvement of autonomic fibers in the polyneuropathy.

In summary, this case shows that GCA can be a paraneoplastic phenomenon of KS and can be complicated by blindness. Several arguments suggest that the polyneuropathy was also paraneoplastic. Oncologists, ophthalmologists, and neurologists should be made aware that GCS and polyneuropathy can be complications of KS and that the combination of GCS and polyneuropathy should prompt a search for malignancy, especially KS.

## Ethical approval

Not applicable.

## Consent to participation

Not applicable.

## Consent for publication

Not applicable.

## Availability of data and material

All data are available from the corresponding author.

## Authors’ contributions

JF: Data curation, Formal analysis, Investigation, Methodology, Project administration, Supervision, Validation, writing revision, Writing - original draft, Writing - review & editing. AF, FS, and CS.

## Funding

None received.

## Declaration of competing interest

The authors declare that the research was conducted in the absence of any commercial or financial relationships that could be construed as a potential conflict of interest.
